# Dimethyl Sulfoxide Attenuates Radiation-Induced Testicular Injury through Facilitating DNA Double-Strand Break Repair

**DOI:** 10.1155/2022/9137812

**Published:** 2022-06-20

**Authors:** Zeze Huang, Renjun Peng, Huijie Yu, Zhongmin Chen, Sinian Wang, Zhengming Wang, Suhe Dong, Wei Li, Qisheng Jiang, Fengsheng Li, Quanmin Li

**Affiliations:** ^1^The Postgraduate Training Base of Jinzhou Medical University (The PLA Rocket Force Characteristic Medical Center), Beijing, China; ^2^Department of Nuclear Radiation Injury and Monitoring, The PLA Rocket Force Characteristic Medical Center, Beijing, China; ^3^Department of Radiotherapy, The PLA Rocket Force Characteristic Medical Center, Beijing, China; ^4^Department of Endocrinology, The PLA Rocket Force Characteristic Medical Center, Beijing, China

## Abstract

The testis is susceptible to ionizing radiation, and male infertility and sexual dysfunction are prevalent problems after whole-body or local radiation exposure. Currently, there is no approved agent for the prevention or treatment of radiation-induced testicular injury. Herein, we investigated the radioprotective effect of dimethyl sulfoxide (DMSO), an organosulfur compound that acts as a free radical scavenger, on testicular injury. Treatment of mice with a single dose of DMSO prior to 5 Gy irradiation restored sex hormones and attenuated the reduction in testis weight. Histological analyses revealed that DMSO alleviated the distorted architecture of seminiferous tubules and promoted seminiferous epithelium regeneration following irradiation. Moreover, DMSO provided quantitative and qualitative protection for sperm and preserved spermatogenesis and fertility in male mice. Mechanistically, DMSO treatment enhanced GFR*α*-1^+^ spermatogonial stem cell and c-Kit^+^ spermatogonial survival and regeneration after radiation. DMSO also alleviated radiation-induced oxidative stress and suppressed radiation-induced germ cell apoptosis *in vivo* and *in vitro*. Additionally, DMSO efficiently reduced DNA damage accumulation and induced the expression of phosph-BRCA1, BRCA1, and RAD51 proteins, indicating that DMSO facilitates DNA damage repair with a bias toward homologous recombination. In summary, our findings demonstrate the radioprotective efficacy of DMSO on the male reproductive system, which warrants further studies for future application in the preservation of male fertility during conventional radiotherapy and nuclear accidents.

## 1. Introduction

Radiotherapy is a common treatment modality for male patients with cancer, such as testicular cancer, prostate carcinoma, and Hodgkin's lymphoma. Ongoing technical advances that allow a more targeted delivery of higher doses of radiation to cancer cells have dramatically improved the outcomes of these diseases, which, however, have made infertility and sexual dysfunction more prevalent and serious [1]. The testis is one of the most radiosensitive organs, and radiation exposure as low as 0.35 Gy causes temporary azoospermia [2]. Radiation-induced testicular injury (RITI) manifests as seminiferous tubule atrophy and reduced the quantity and quality of sperm, which results in temporary or permanent infertility. Radioprotectors are agents that are administered prophylactically to prevent radiation-induced injuries. To date, no radioprotector specifically designed for male cancer patients has been approved to protect against RITI [3]. In addition, such agents would be useful for workers occupationally exposed to ionizing radiation (IR), first responders, and civilians in the case of nuclear accidents. Therefore, highly effective radioprotectors with fewer side effects and affordable price are urgently needed for RITI.

Spermatogenesis, which takes place within the testis seminiferous epithelium, is a tightly controlled multistep process of male germ cell proliferation and maturation from spermatogonial stem cells (SSCs) to spermatozoa. SSCs are a rare population of germ cells with the potential to self-renew and differentiate, sustaining continuous production of the male germline [4]. For low linear energy transfer (LET) radiations involving X and gamma ray, the initiation of RITI is mainly mediated through free radicals produced by the radiolysis of cellular water after radiation exposure. These free radicals are reactive oxygen species (ROS), such as ^**·**^OH and O_2_^**·-**^, that attack DNA bases, oxidate proteins, and peroxidate membrane lipids, disturbing the metabolism, proliferation, and differentiation of germ cells [3]. DNA double-strand breaks (DSBs) are mostly induced by ^**·**^OH and are the most serious type of damage among IR-induced genome toxicities [5]. DSBs in SSCs and progenitors initiate a coordinated signaling network that recognizes exposed DNA lesions, undergoes cell cycle arrest, activates DSB repair, or induces cell death [6]. Accumulating evidence has demonstrated that IR-induced germ cell loss is mainly mediated via apoptosis, with actively proliferating spermatogonia being the most susceptible, followed by SSCs [7]. Radiation-induced depletion of SSCs impairs germline recovery; thus, surviving SSCs are fundamental for regeneration following radiation exposure.

Because of the role of ROS in the pathogenesis of RITI, antioxidants with the capability to scavenge free radicals are considered strong candidates for radioprotectors. Dimethyl sulfoxide (DMSO) is capable of reacting with hydroxyl radicals (^**·**^OH) to generate methyl radicals (^**·**^CH3) with lower reactivity, which makes it a widely used radical scavenger [8]. DMSO exhibits therapeutic benefits in many human diseases [9–11]. Moreover, DMSO can exert radioprotective effects by facilitating DNA DSB repair [12]. Recently, we reported that DMSO confers protection to hematopoietic injury and oral mucositis induced by lethal dosages of radiation [13, 14]. In the present study, we investigated whether DMSO could protect mice from testicular injury induced by IR. Our results demonstrated the radioprotective efficacy of DMSO against RITI. Specifically, DMSO reduces DNA damage accumulation and germ cell apoptosis, thus facilitating spermatogenesis recovery postirradiation by preserving the SSC pool.

## 2. Materials and Methods

### 2.1. Mice

Male C57BL/6J mice were purchased from SPF Biotechnology Co., Ltd. (Beijing, China). All mice were used at approximately 6-8 weeks of age, weighing 22~24 g. Acidified water and standard chow were provided. The procedures for animal experimentation were approved by the Ethics Committee of Chinese People's Liberation Army (PLA) Rocket Force Characteristic Medical Center and were performed in accordance with the Guide for the Care and Use of Laboratory Animals.

### 2.2. Cells and Cell Culture

The spermatocyte cell line GC-2 was purchased from Procell Life Science & Technology Co., Ltd. (Wuhan, China). Cells were cultured in high-glucose Dulbecco's modified Eagle's medium (DMEM; 8121431, Gibco, China) supplemented with 10% fetal bovine serum (aq 45586256, Analysis Quiz, China) and 1% penicillin-streptomycin (2321128, Gibco, China) and incubated in a humidified incubator at 37°C under 5% CO_2_.

### 2.3. Irradiation and DMSO Administration

X-rays produced by a precision linear accelerator (VWAT Elekta, Sweden) in the Radiation Centre (PLA Rocket Force Characteristic Medical Center, Beijing) were used as radiation sources. Mice were subjected to 5 Gy total-body irradiation (TBI) at a dose rate of 4 Gy/min. Mice were treated with vehicle (saline) or DMSO (M08G704, Alfa Aesar, China) at a dose of 6 g/kg by intraperitoneal injection 1 h prior to irradiation. For the *in vitro* experiment, GC-2 cells were subjected to irradiation at a dose rate of 4 Gy/min and cultured with or without 0.5% DMSO (*v*/*v*) for 2 h prior to irradiation.

### 2.4. Assessment of Male Fertility

The fertility of experimental mice was evaluated 60 days post-IR. 6 males from each group were individually housed with three sexually mature virgin females (*n* = 18 per group) for successive 60 days. Once pregnant, females were removed to individual cage. Immediately after parturition, the number of the litter and the interval time between the date of mating and childbirth were recorded. Pregnant females (%) were calculated as the ratio of pregnant female mice to the total female mice in each group (*n* = 18). Litter size was calculated as the means of litter of bred females among the same group.

### 2.5. Hormone Assays

Mouse blood samples were extracted from the retroorbital sinus and centrifuged at 3500 g for 20 minutes, and serum samples were collected and stored at -80°C. The serum concentrations of testosterone, luteinizing hormone (LH), follicle-stimulating hormone (FSH), and gonadotropin-releasing hormone (GnRH) were determined by ELISA. All ELISA kits were purchased from Shanghai Enzyme-Linked Biotechnology Co., Ltd. (mlbio, China) and used according to the manufacturer's instructions.

### 2.6. Histological Analysis

The right testis of mice was dissected out on days 15, 30, 45, 60, and 120 after irradiation, and the caput of the epididymis is harvested on day 60 after irradiation. Tissues were fixed immediately in Bouin's solution (G1121, Servicebio, China), embedded in paraffin, and sectioned. Histological analysis was performed following hematoxylin and eosin (H&E) staining. The diameter and germinal epithelium thickness (from the basement membrane to lumen) of seminiferous tubules were measured by using a digital imaging microscope (DMI8A, Leica, Germany) with Leica LAS X software. Quantitative results were counted with thirty random seminiferous tubules from each section. To observe the regenerated mature sperm, the efferent duct of the caput of the epididymis was examined.

### 2.7. Sperm Count and Morphology

Animals were scarified on days 15, 30, 45, 60, and 120 after irradiation. Both sides of the cauda epididymis were removed and minced in an Eppendorf tube containing 1 mL HEPES (C0215, Beyotime, China) and incubated for 10 min in a 37°C water bath. 10 *μ*L of supernatant was transferred to a cell counting plate to count sperm under a light microscope (Olympus, Japan) after incubation. To observe the sperm morphology after irradiation, slides were prepared on the 15^th^, 30^th^, 45^th^, 60^th^, and 120^th^ day post-IR. Another 50 *μ*L of supernatant for smearing was fixed in 80% ethanol and stained with 1% eosin. For each mouse, at least 200 sperm were examined using a light microscope at 1000x magnification for normal and abnormal (hook-less, banana-like, amorphous, folded, short tail, two-tail, and two-head) forms [15, 16].

### 2.8. Immunohistochemistry and Immunofluorescence

The detailed methods of immunostaining for DDX4/MVH, Ki67, cleaved caspase-3, c-Kit, GFR*α*-1, and *γ*-H2AX are described in Supplementary Materials.

### 2.9. Cell Count Kit-8 (CCK-8) and Colony Assay

GC-2 cells were plated in 96-well plates at a density of 1 × 10^3^ cells/well, cultured overnight, and subsequently treated with 0.5% DMSO (*v*/*v*) for 2 h, followed by an escalating dose of irradiation (0-14 Gy). CCK-8 (AQ308-500t, China) was used to assess GC-2 cell viability. The optical density (OD) value was measured using a microplate reader at a wavelength of 450 nm.

For standard colony assays, GC-2 cells were seeded in triplicate in 6-well plates at densities of 500 cells/well. After overnight culture, the cells were pretreated with 0.5% DMSO (*v*/*v*) for 2 h, followed by 10 and 12 Gy radiation. The cells were incubated for another 7 days to allow for colony formation and subsequently stained with crystal violet. Pictures of the plates were acquired, and the number of colonies was quantified.

### 2.10. Terminal Deoxynucleotidyl Transferase dUTP Nick-End Labeling (TUNEL) Assay

Germ cell apoptosis was detected in testicular paraffin sections by a TUNEL assay kit (C1098, Beyotime, China) according to the manufacturer's instructions. The number of TUNEL-positive cells was counted with thirty random seminiferous tubules from each section.

### 2.11. Cellular Apoptosis and ROS Assay

Apoptosis of GC-2 cells was measured using the Annexin V and Dead Cell kit (MCH100105; Luminex). Briefly, 1 × 10^5^-1 × 10^6^/mL single-cell suspension was prepared in advance, and 100 *μ*L Muse Annexin V and Dead Cell Reagent was added to each tube and then incubated for 20 minutes at room temperature protected from light. Then, the sample was analyzed by using a Guava® Muse Cell Analyzer.

ROS levels in GC-2 cells were assayed by using a Muse Oxidative Stress kit (MCH 100111, Luminex). Briefly, cell samples (1 × 10^5^/mL) and oxidative stress reagent solution were prepared with 1X assay buffer, mixed thoroughly, and then incubated for 30 minutes at 37°C protected from light. The proportion of ROS-positive cells was measured by using a Guava® Muse Cell Analyzer.

### 2.12. Superoxide Dismutase (SOD) and Malondialdehyde (MDA) Assay

The mouse testis was collected 6 h after 5 Gy irradiation and homogenized in precooled PBS, and the supernatant was centrifuged to obtain the assay sample. SOD was measured by using a total superoxide dismutase assay kit (S0101-M, Beyotime, China) according to the manufacturer's instructions. For the determination of MDA, the lipid peroxidation MDA assay kit (S0131-M, Beyotime, China) was used following the manufacturer's instructions.

### 2.13. Western Blot

Western blot was performed by standard procedures. BRCA1 antibody (ab191042, Abcam, 1 : 1000), *γ*-H2AX (phospho S139) antibody (ab11174, Abcam, 1 : 2000), p-BRCA1 (S1524) antibody (9009T, Cell Signaling Technologies, 1: 1000), RAD51 (D4B10) antibody (8875T, Cell Signaling Technologies, 1 : 1000), and Vinculin antibody (A01207, BOSTER, 1 : 1000) were used. Finally, the results were visualized using a Bio-Rad ChemiDoc MP imaging system (California, USA).

### 2.14. Statistical Analysis

GraphPad Prism 8.0 (GraphPad Software, San Diego, California) was used for statistical analysis. Data were presented as the means ± standard deviation (SD). Comparisons between groups were performed using an unpaired two-tailed Student's *t*-test. Asterisks represent the *p* values as follows: ^∗^*p* < 0.05, ^∗∗^*p* < 0.01, ^∗∗∗^*p* < 0.001, and ^∗∗∗∗^*p* < 0.0001. *p* < 0.05 was considered statistically significant.

## 3. Results

### 3.1. DMSO Preserves Fertility in Mice after Irradiation

To evaluate the effect of DMSO on fertility after irradiation, mice were intraperitoneally injected with 6 g/kg DMSO one hour prior to 5 Gy TBI, and irradiated male mice were mated with female mice 60 days later. All female mice in the unirradiated group gave birth to mice, whereas only 50.0% of female mice in the irradiated-alone group became pregnant. Compared with the nonirradiated group, the time interval from mating to childbirth of female mice from the irradiated-alone group was extended by 1.5 times, and the litter size was reduced by 46.7%. In contrast, DMSO pretreatment significantly preserved the fertility of irradiated mice; a total of 83.3% of female mice were pregnant, with a shorter conception time and a larger litter size (Table 1).

### 3.2. DMSO Promotes Sex Hormone Recovery after Irradiation

Sex hormones, such as testosterone, FSH, LH, and GnRH, play an essential role in sexual function and spermatogenesis [17, 18]. The serum concentrations of these hormones in mice were determined 60 days after irradiation by ELISA. The results showed a remarkable decline in the abovementioned hormones after 5 Gy radiation, whereas DMSO pretreatment increased the serum levels of testosterone, LH, and FSH. DMSO-treated mice had lower serum concentrations of GnRH than vehicle-treated mice after radiation, indicating the negative feedback regulation of sex hormones (Table.  2). Similar sex hormone alterations were observed 45 days after irradiation (Supplemental Table S1).

### 3.3. DMSO Ameliorates Radiation-Induced Testicular Damage

**Figure 1:**
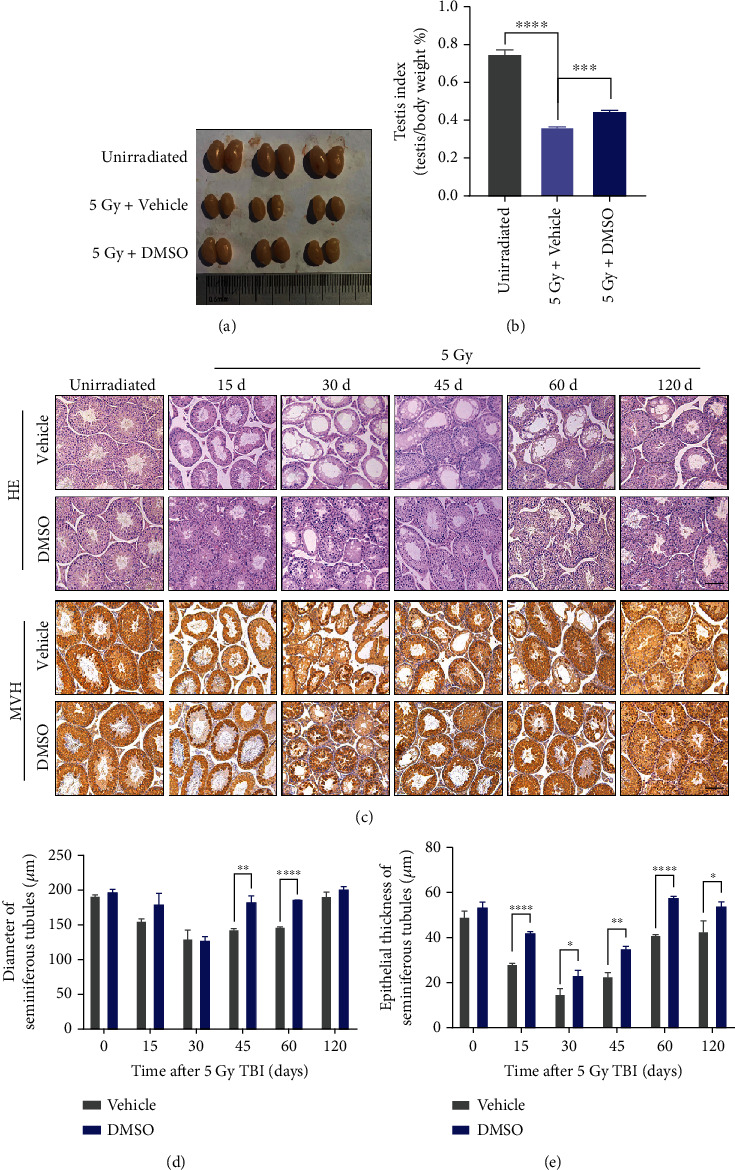
DMSO alleviates radiation-induced testicular injury and enhances seminiferous epithelium regeneration after irradiation. (a) The image of harvested testis and (b) the calculated testis index at 60 d post-IR. (c) Representative images of the H&E- and MVH- (DDX4) stained testis at the indicated days post-IR. Scale bar = 100 *μ*m. (d, e) Quantitative analysis of the diameter and the epithelial thickness of seminiferous tubules at various time points post-IR. Error bars indicate mean ± SD (*n* = 3). ^∗^*p* < 0.05, ^∗∗^*p* < 0.01, and ^∗∗∗^*p* < 0.001. Student's *t*-tests were used to determine statistical significance in (a), (d), and (e).

The testis showed clear atrophy (Figure 1(a)), and the relative index (testis/body weight) declined by 51.4% at day 60 after irradiation compared with that in the unirradiated control. Meanwhile, DMSO pretreatment significantly suppressed the atrophy of the testis, reaching 59.6% of the unirradiated controls (Figure 1(b)). Histological analysis showed that 5 Gy irradiation resulted in severe testicular atrophy, with disorganization of spermatogenic cells and disappearance of mature spermatozoa in the lumen at day 30 post-IR. Thereafter, the damaged testis gradually recovered, and atrophied spermatogenic epithelium was still visible 120 days after irradiation. Mice pretreated with DMSO exhibited comparatively normal testicular structure with regular cell arrangement and less spermatogenic cell loss; these mice recovered faster than the irradiated group (Figure 1(c)). MVH is a pan germ cell expression marker [19]. We observed that MVH-positive cells in the seminiferous tubules were reduced to a minimum and even disappeared after 30 days of irradiation, whereas DMSO-pretreated mice displayed more MVH-positive cells (Figure 1(c)). In addition, DMSO accelerated the recovery of the seminiferous tubules and epithelium thickness to the unirradiated level (Figures 1(d) and 1(e)). These results strongly demonstrate that DMSO enhances testicular reconstruction after radiation.

### 3.4. DMSO Promotes Recovery of Sperm Counts and Reduces Abnormalities after Irradiation

**Figure 2:**
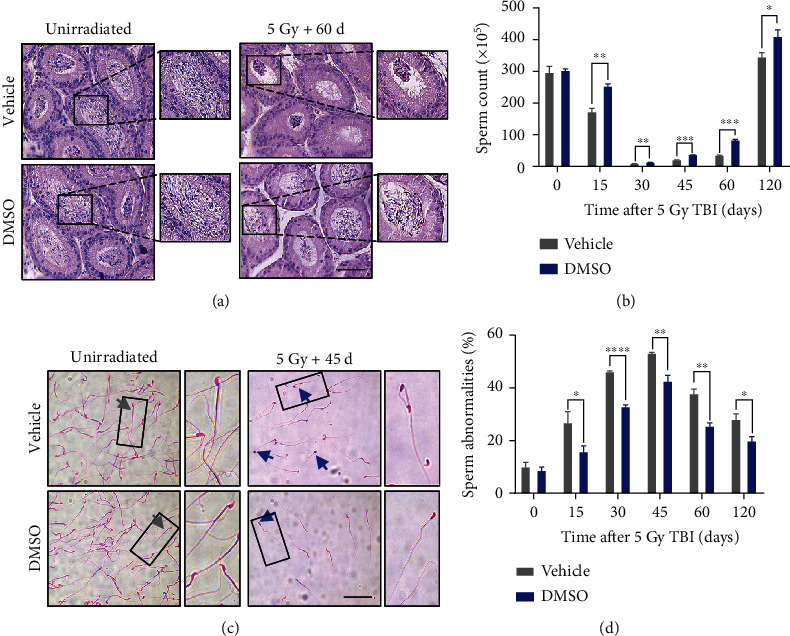
DMSO reverses the decline of sperm quantity and quality after irradiation. (a) Representative images of the H&E-stained epididymis at 60 d post-IR. Scale bar = 50 *μ*m. (b) Sperm counts at the indicated time points after irradiation. (c) Representative images of eosin-stained sperms before or 45 d after irradiation; normal and abnormal sperm are indicated by gray and blue arrows, respectively (scale bar = 50 *μ*m). (d) Percent of abnormal sperm at the indicated time points after irradiation. Error bars indicate mean ± SD (*n* = 3). ^∗^*p* < 0.05, ^∗∗^*p* < 0.01, and ^∗∗∗^*p* < 0.001. Student's *t*-tests were used to determine statistical significance in (b) and (d).

The maintenance of male fertility is directly dependent on the continuous production of mature spermatozoa, which are released from the seminiferous tubule lumen and transported to the epididymis [4]. Two months after irradiation, a few spermatozoa could be seen in the epididymis, while spermatozoa started to abundantly emerge in those from DMSO-treated mice (Figure 2(a)). We further carried out sperm counts in the epididymis. At days 30, 45, and 60 post 5 Gy irradiation, the sperm sharply decreased to 3.0%, 8.0%, and 12.0% of the nonirradiated level, respectively, while the sperm counts in DMSO-treated mice increased by 1.8-fold, 1.7-fold, and 2.3-fold compared with the vehicle-treated mice at these time points, respectively (Figure 2(b)). Exposure to radiation gave rise to decreased sperm counts as well as increased sperm abnormalities [20]. Sperm abnormalities including hook-less, banana-like, amorphous, folded, short-tail, two-tail, and two-head spermatozoa were counted [15, 16]. The proportion of sperm abnormalities reached its peak 30-45 days after irradiation, while DMSO remarkably reduced the sperm abnormalities compared with the control group (Figures 2(c) and 2(d)). These results indicate that DMSO provides quantitative and qualitative protection for spermatozoa.

### 3.5. DMSO Promotes the Survival and Proliferation of Germ Cells after Radiation *In Vivo* and *In Vitro*

**Figure 3:**
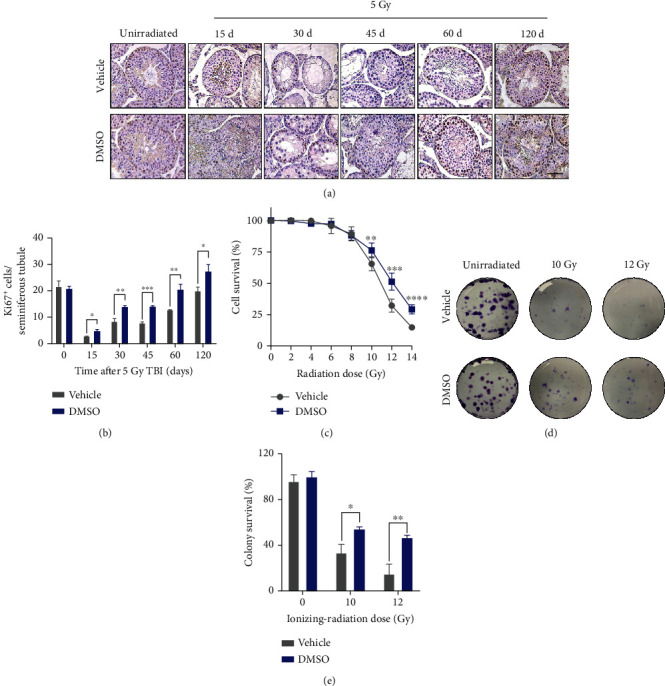
DMSO radioprotects germ cells *in vivo* and *in vitro*. (a) Representative images of the Ki67-stained testis and (b) quantitative analysis are shown at the indicated days post-IR. Scale bar = 50 *μ*m. (c) Cell survival was detected by CCK-8 after irradiation at various dosages (0-14 Gy). Data from all irradiated samples were normalized to unirradiated samples, and percentages of viable cells were plotted. (d) Representative images of cell plates showing colonies derived from DMSO- or vehicle-treated GC-2 cells either left unirradiated or irradiated with 10 Gy and 12 Gy. (e) Quantitative analysis showing the percentages of surviving colonies. Error bars indicate mean ± SD (*n* = 3). ^∗^*p* < 0.05, ^∗∗^*p* < 0.01, ^∗∗∗^*p* < 0.001, and ^∗∗∗∗^*p* < 0.0001. Student's *t*-tests were used to determine statistical significance in (b), (c), and (e).

The continuous production of spermatozoa results from the proliferation and differentiation of spermatogenic cells. We used Ki67 staining to determine the spermatogenesis after irradiation. The results showed that Ki67-positive cells declined sharply at day 15 after irradiation and recovered gradually thereafter. In contrast, more Ki67-positive cells were observed in the testis of DMSO-treated mice (Figures 3(a) and 3(b)). We next sought to determine whether DMSO could directly protect spermatogenic cells following irradiation *in vitro*. GC-2 cells were pretreated with 0.5% DMSO (*v*/*v*) and subjected to escalating dosages of irradiation (0-14 Gy). A significant increase in radioresistance was observed in the DMSO-treated group (Figure 3(c)). Further colony-forming assays revealed that, after 10 Gy and 12 Gy irradiation, the total number of colonies pretreated with DMSO was 1.6-fold and 3.1-fold higher, respectively, than that of the vehicle group on average (Figures 3(d) and 3(e)). DMSO exerted similar radioprotection to irradiated GC-1 cells (Supplemental Figure 1) whereas DMSO pretreatment showed no protective effect on mouse colon carcinoma cell (CT-26) survival after irradiation (Supplemental Figure 2).

### 3.6. DMSO Protects Spermatogonial Stem Cells and Spermatogonia from Ionizing Radiation-Induced Injury

**Figure 4:**
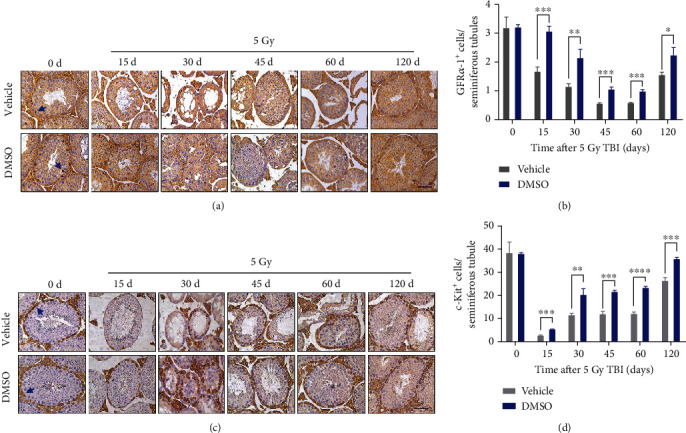
DMSO improves spermatogonial stem cells and spermatogonia survival after irradiation. (a) Representative images of the GFR*α*-1-stained testis within 120 d after irradiation; GFR*α*-1-positive cells are indicated by blue arrows. (b) Quantitative analysis is shown at the indicated days post-IR. Scale bar = 50 *μ*m. (c) Representative images of the c-Kit-stained testis and (d) quantitative analysis are shown at the indicated days post-IR. Scale bar = 50 *μ*m. Error bars indicate mean ± SD (*n* = 3). ^∗^*p* < 0.05, ^∗∗^*p* < 0.01, and ^∗∗∗^*p* < 0.001. Student's *t*-tests were used to determine statistical significance in (b) and (d).

SSCs play a vital role in the reconstruction of testicular tissue upon damage [4]. Transplanted glial cell line-derived neurotrophic factor family receptor *α*-1^+^ (GFR*α*-1^+^) cells have the capacity to reconstruct the spermatogenic epithelium, and GFR*α*-1^+^ cells persist in lineage-marked cell clones within the testis [21]. Hence, SSCs are included in the GFR*α*-1^+^ cell pool. We observed that GFR*α*-1^+^ cells were located in the basal layer of the seminiferous epithelium, with approximately 3 positive cells in each lumen at a steady state (Figure 4(a)). GFR*α*-1^+^ cells were gradually reduced to their minimum at day 60 and returned to half of their preirradiation level at day 120 after irradiation. Compared to the control, DMSO dramatically inhibited the loss of GFR*α*-1^+^ cells and promoted the relative recovery of these cells (Figure 4(b)). c-Kit-expressing spermatogonia act as transit-amplifying progenitors, which undergo a process of meiosis and maturation to produce spermatozoa [21]. As shown in Figure 4(c), we observed that loss of c-Kit^+^ cells after radiation was more rapid and severe than that of GFR*α*-1^+^ cells. DMSO pretreatment also potently reduced radiation-induced depletion of c-Kit^+^ cells and promoted recovery at later phases (Figure 4(d)).

### 3.7. DMSO Suppresses Irradiation-Induced Apoptosis of Germ Cells

**Figure 5:**
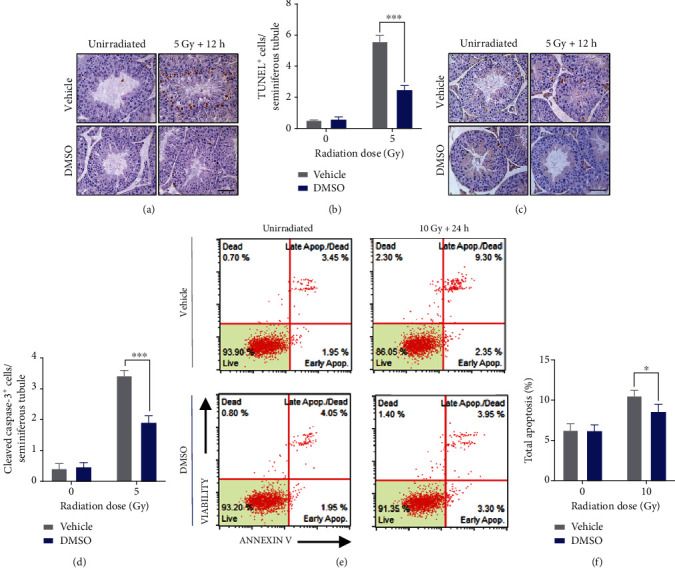
DMSO suppresses radiation-induced germ cell apoptosis *in vivo* and *in vitro*. (a) Representative images of the TUNEL-stained testis 12 h post-IR, scale bar = 50 *μ*m. (b) Quantification of TUNEL-positive cells. (c) Representative images of the cleaved caspase-3-stained testis 12 h post-IR, scale bar = 50 *μ*m. (d) Quantification of cleaved caspase-3-positive cells. (e) Representative flow cytometric analysis of apoptosis in GC-2 cells 24 h post-IR. (f) Percentages of Annexin V-positive GC-2 cells. Error bars indicate mean ± SD (*n* = 4). ^∗^*p* < 0.05, ^∗∗∗^*p* < 0.001. Student's *t*-tests were used to determine statistical significance in (b), (d), and (f).

Radiation-induced testicular toxicity partially results from massive germ cell apoptosis [22]. We performed TUNEL and cleaved caspase-3 (CC3) staining in testicular sections 12 h after 5 Gy TBI. Irradiation increased the number of TUNEL^+^ cells by 10.3 times, while elevated apoptotic cells were inhibited by 55.9% in the DMSO-treated mouse testis (Figures 5(a) and 5(b)). Similar results were found in the quantification of apoptotic CC3^+^ cells (Figures 5(c) and 5(d)). To further clarify the impact of DMSO on germ cell apoptosis, GC-2 cells were subjected to 10 Gy irradiation, stained with FITC-Annexin V/7-AAD, and analyzed by flow cytometry (Figure 5(e)). DMSO pretreatment significantly reduced the proportion of Annexin V-positive cells in comparison with the vehicle group (Figure 5(f)). We observed similar results in GC-1 cells (Supplemental Figure 3). These *in vivo* and *in vitro* data demonstrate that DMSO protects RITI by suppressing radiation-induced apoptosis in the testis.

### 3.8. DMSO Reduces Irradiation-Induced Oxidative Stress *In Vivo* and *In Vitro*

**Figure 6:**
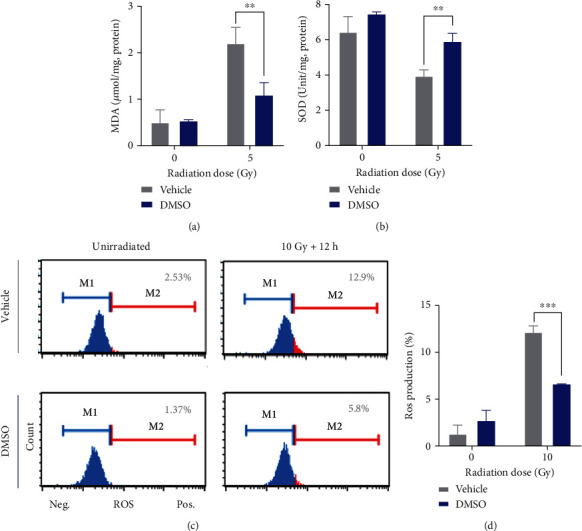
DMSO alleviates radiation-induced oxidative stress *in vivo* and *in vitro*. (a) Malondialdehyde (MDA) levels and (b) the activity of superoxide dismutase (SOD) in the testis at 6 h post-IR were determined. (c) Representative flow cytometric analysis of ROS in GC-2 cells 12 h post-IR. (d) Quantification of percentages of the ROS-positive GC-2 cells. Error bars indicate mean ± SD (*n* = 3). ^∗∗^*p* < 0.01, ^∗∗∗^*p* < 0.001. Student's *t*-tests were used to determine statistical significance in (a), (b), and (d).

Oxidative stress was evaluated by measuring lipid peroxides and antioxidant enzyme SOD activity. Irradiation (5 Gy) led to a significant elevation in lipid peroxide contents (MDA) and a decline in SOD activity in testicular tissues. However, irradiated mice pretreated with DMSO exhibited equivalent levels of both MDA and SOD as the unirradiated controls (Figures 6(a) and 6(b)). We next determined whether DMSO could attenuate oxidative stress *in vitro*. GC-2 cells were subjected to 10 Gy irradiation, and ROS production was measured 12 hours later by flow cytometry (Figure 6(c)). Compared with the irradiated control group, the proportion of ROS-positive cells in the DMSO-pretreated group decreased by 50% (Figure 6(d)). We also observed similar results in GC-1 cells (Supplemental Figure 4).

### 3.9. DMSO Facilitates DNA DSB Repair and Decreases DNA Damage Accumulation

**Figure 7:**
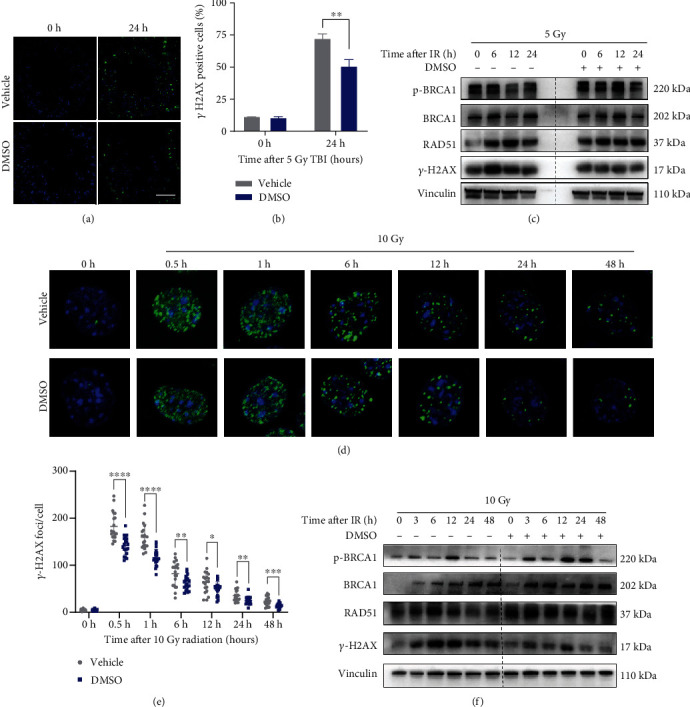
DMSO facilitates DNA damage repair with a bias toward homologous recombination. (a) The testis was harvested 12 h after irradiation and stained with *γ*-H2AX. Images were taken with a confocal microscope; *γ*-H2AX foci are shown (b). (c) The testis was harvested at 0 h, 6 h, 12 h, and 24 h post-IR. WB analysis was performed to examine the protein levels of *γ*-H2AX, RAD51, BRCA1, and p-BRCA1. Vinculin was used as a loading control. (d) GC-2 cells were harvested at 0 h, 0.5 h, 6 h, 12 h, 24 h, and 48 h after irradiation and stained with *γ*-H2AX. *γ*-H2AX foci are shown in green, and DAPI is colored in blue. Images were taken with a confocal microscope. (e) Quantitative analysis of *γ*-H2AX foci in GC-2 cells. (f) GC-2 cells were harvested at 0 h, 3 h, 6 h, 12 h, 24 h, and 48 h after irradiation, and WB analysis was performed to examine the protein levels of *γ*-H2AX, RAD51, BRCA1, and p-BRCA1. Vinculin was used as a loading control. Error bars indicate mean ± SD (*n* = 3). ^∗^*p* < 0.05, ^∗∗^*p* < 0.01, ^∗∗∗^*p* < 0.001, and ^∗∗∗∗^*p* < 0.0001. Student's *t*-tests were used to determine statistical significance in (b) and (e).

*γ*-H2AX foci at later phases following radiation are considered surrogates for unresolved DSBs [23]. To clarify whether DMSO can decrease DNA damage, we examined unrepaired DNA DSBs in the testis by *γ*-H2AX staining (Figure 7(a)). As expected, DMSO dramatically decreased the proportion of *γ*-H2AX-positive cells in the seminiferous epithelium area (Figure 7(b)). Similarly, immunoblotting also showed decreased *γ*-H2AX expression in the testis from DMSO-treated mice. BRCA1 and RAD51 are homologous recombination- (HR-) specific repair mediators that are incorporated into discrete IR-induced repair foci (IRIFs), which are rapidly produced at DSB sites [5]. Pretreatment with DMSO showed elevated expression of phospho-BRCA1, BRCA1, and RAD51 in the mouse testis following irradiation (Figure 7(c)). DSBs are also programmed to occur during meiosis [24]. To exclude the interference of these physiological DSBs with the results, GC2 cells were irradiated, and the time-based kinetics of *γ*-H2AX foci were then analyzed. The results revealed that the number of *γ*-H2AX foci in DMSO-treated cells was significantly lower than that in vehicle-treated cells (Figure 7(d)). Similarly, we found that DMSO increased the expression of phospho-BRCA1, BRCA1, and RAD51 and accelerated the dissolution kinetics of *γ*-H2AX after radiation exposure (Figure 7(e)). We provided *in vivo* and *in vitro* evidence that DMSO can potently decrease radiation-induced DNA damage and promote HR-mediated repair.

## 4. Discussion

Although the efficacy of radiotherapy is a top priority for cancer treatment, the potential testicular injury induced by ionizing radiation may cause considerable distress to male patients. Radioprotectors to minimize or reverse testicular injury are urgently needed. In the present study, we provided evidence that DMSO preserves spermatogenesis and fertility in male mice after irradiation exposure. First, DMSO promotes the regeneration of the testicular seminiferous epithelium (Figure 1); second, DMSO provides quantitative and qualitative protection for spermatozoa (Figure 2); and third, DMSO restores gonadal hormones after irradiation (Table 2). All of results may explain the potent protection of DMSO against the fertility impairment induced by ionizing radiation in male mice (Table 1).

DMSO suppresses radiation-induced oxidative stress and facilitates DNA damage repair by promoting expression of BRCA1 and RAD51, thereby reducing DNA damage accumulation in testicular cells. DMSO further inhibits apoptosis and enhances spermatogonial stem cells and spermatogonial survival, which promotes the regeneration of the seminiferous epithelium and preserves the fertility of male mice following radiation.

To date, there is no approved radioprotector for RITI [3]. Amifostine (WR2721) has been approved for xerostomia associated with radiotherapy. However, the further clinical application of amifostine in RITI is limited by its side effects, such as hypertension, nausea, and cytotoxicity to spermatogonial stem cells [25, 26]. Entolimod (CBLB502), a Toll-like receptor 5 agonist derived from Salmonella flagellin, has been shown to alleviate RITI [16], which might trigger unexpected toxic immunogenic reactions [27]. Hydrogen-rich saline has potential as a novel radiation countermeasure without known toxic side effects; however, its narrow protective time frame (5 min) before radiation has restricted further applications [28]. Other reported herbal medicines with radioprotective effects need repeated administration [29, 30], which may reduce patient compliance and cannot be used in nuclear emergency rescue. Our study clearly shows that a single dose of DMSO within a short period of time before radiation (1 h) is effective at promoting spermatogenesis and reducing the abnormal percentage of sperm even 120 days after exposure. This is particularly important because radiation therapy for male patients is started shortly after cancer diagnosis, and fertility must be rapidly and effectively preserved at the same time [31]. In addition, DMSO also has the advantages of low systemic toxicity [31, 32], stability at room temperature, and low price. Therefore, DMSO is a promising candidate for the further development of human use in a variety of settings, including radiotherapy, occupational radiation protection, and nuclear accidents.

Since SSCs are the basis for the regeneration of the seminiferous epithelium after injury, protecting SSCs should be a priority for developing novel radioprotectors against RITI. Here, we stained the testis section for the stem cell marker GFR*α*-1 and prove that DMSO retains SSCs in the testis after radiation exposure. In addition, SSCs and spermatogonial cells (marked by c-Kit) decreased by 92.0% and 48.0%, respectively, 15 days after irradiation, which is consistent with the fact that SSCs are more resistant to radiation [7]. Even 120 days after radiation exposure, SSCs and spermatogonia still failed to return to normal levels, suggesting long-term defects in spermatogenesis. In contrast, mice in the DMSO-treated group exhibited more SSCs and progenitors, suggesting that DMSO mitigated both acute testicular injury and long-term spermatogenesis suppression in mice after radiation (Figure 4). Apoptosis is believed to be the major mechanism underlying massive cell loss in radiosensitive tissues [22]. Here, we provided *in vivo* and *in vitro* evidence that DMSO markedly inhibits apoptosis in radiated spermatogenic cells (Figure 5), which partly explains the reduced spermatogenic cell loss in seminiferous tubules after irradiation.

DSBs are life-threatening lesions. Failure to repair DSBs, or their misrepair, may result in cell death or genome instability with persistent DNA damage. Several groups have reported that DMSO can ameliorate oxidative stress by scavenging ROS [32]. Correspondingly, we found that DMSO decreases MDA levels and induces the expression of the antioxidant enzyme SOD in the testis of irradiated mice (Figure 6). We also observed that DMSO dramatically suppressed the expression of gamma-H2AX (an indicator of DSB damage) at the early phase in irradiated testis (6 h) and GC-2 cells (≤6 h). These results together indicate that DMSO directly inhibits the toxicity of ROS. Chan et al. [33] reported that 0.1 M DMSO has a limited scavenging effect in hypoxia but still reduced all types of DNA damage in anoxic cells. Other studies show that DMSO can facilitate DNA DSB repair at lower nontoxic concentrations (0.5%, *v*/*v*, 64 mM) [12, 14], indicating that the radioprotective mechanism needs to be further explored. Tunçer et al. [34] reported that low-dose DMSO (0.1%-1.5%, *v*/*v*) induces a number of changes in all macromolecules, especially in proteins and nucleic acids. DMSO affects protein secondary structures, with more *β*-sheet content, which may affect the activity of proteins. Besides, DMSO reduces nucleic acid level accompanied by the formation of Z-DNA. This alternate DNA form may be related to the specific actions of DMSO on gene expression. We provided further evidence that DMSO can promote the expression of HR-related proteins (i.e., RAD51 and BRCA1) in the irradiated mouse testis and cultured cells (Figure 7), which allows for the generation of spermatozoa populations with high-fidelity DNA required to maintain genomic stability. The role and potential mechanisms of increased HR dependence on DMSO-mediated testicular radioprotection warrant further experimentation.

The main potential risk of systemic delivery of DMSO is the radioprotection of tumor cells. Homologous recombination deficiency (HRD) is a common characteristic of many tumors. Nguyen et al. analyzed the data of 5122 tumor patients and found that HRD is most frequent in ovarian and breast cancer, followed by pancreatic and prostate cancer [35]. As reported, low-concentration DMSO (64 mM) exhibited no radioprotective effects in xrs5 cells with DNA damage repair defects [12]. Our previous study demonstrated that DMSO protects against normal tissue without tumor protection in CAL-27 or MLL-AF9 xenograft mice [13, 14]. In line with these studies, we did not observe any protective ability of DMSO (64 mM) in CT-26 colon cancer cells *in vitro*, regardless of whether it was combined with irradiation. In contrast, Hashimoto et al. reported the radioprotection of DMSO (0.25 M) to EMT6 sarcoma cells and HSG human salivary gland tumor cells [36], and Zwicker et al. found that colon carcinoma WiDr cells treated with DMSO (0.28 M) showed increased clonogenic survival [37]. This contradiction might be derived from the variations in DMSO concentration, irradiation dose, and cell line used. *In vivo* mouse models should be used in further studies to test whether DMSO treatment provides selective radioprotection to normal cells.

## 5. Conclusions

**Figure 8:**
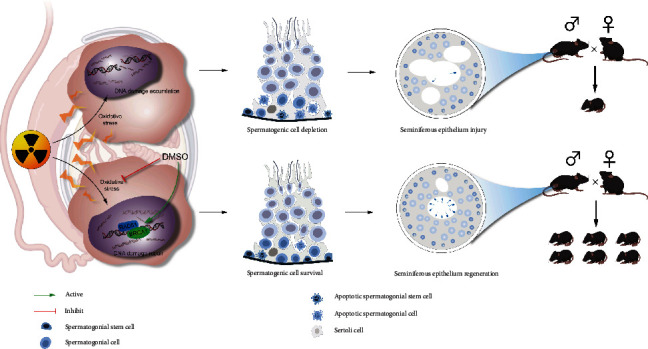
Proposed mechanisms underlying the radioprotective effect of DMSO in the mouse testis.

In summary, the results obtained herein demonstrated for the first time that DMSO plays multiple roles in protecting against testicular injury induced by ionizing radiation. DMSO pretreatment restores sex hormones, promotes reconstruction of testis tissue, and reverses the decrease in sperm quantity and quality after irradiation, which ultimately contributes to the preservation of spermatogenesis and fertility. Mechanistically, we provided *in vivo* and *in vitro* evidence that radiation-induced oxidative stress and apoptosis are both suppressed by DMSO, which may further enhance SSCs survival and regeneration after radiation. Interestingly, DMSO efficiently reduced DNA damage accumulation and induced the expression of phosph-BRCA1, BRCA1, and RAD51 proteins, indicating that DMSO facilitates DNA damage repair with a bias toward homologous recombination (Figure 8). Our work demonstrates the radioprotective efficacy of DMSO on the male reproductive system and warrants further research for future applications in the preservation of male fertility during conventional radiotherapy and nuclear accidents.

## Figures and Tables

**Table 1:** Fertility assessment 60 days after total body irradiation.

Group	Pregnant female (%)	Time interval from mating to childbirth (days)	Litter size
Unirradiated	100.0	23.5 ± 5.0	8.3 ± 1.4
5 Gy+vehicle	50.0	36.2 ± 5.4^###^	4.4 ± 1.9^###^
5 Gy+DMSO	83.3	30.7 ± 6.1^∗^	7.2 ± 1.5^∗∗^

Female mice (*n* = 18) for each group; values are expressed as mean ± SD. ^###^*p* < 0.001 for 5 Gy+vehicle vs. unirradiated. ^∗^*p* < 0.05 for 5 Gy+DMSO vs. 5 Gy+vehicle; ^∗∗^*p* < 0.05 for 5 Gy+DMSO vs. 5 Gy+vehicle.

**Table 2:** The effect of DMSO on serum concentrations of sex hormones 60 days postirradiation.

Group	Testosterone (ng/mL)	LH (mIU/mL)	FSH (mIU/mL)	GnRH (mIU/mL)
Unirradiated	53.7 ± 8.5	15.0 ± 1.8	147.2 ± 8.0	229.8 ± 19.5
DMSO	46.9 ± 3.2	13.7 ± 0.8	140.0 ± 7.0	193.0 ± 13.9
5 Gy+vehicle	18.6 ± 2.4^##^	5.1 ± 0.5^###^	50.6 ± 2.8^####^	113.5 ± 14.7^##^
5 Gy+DMSO	27.9 ± 2.9^∗^	8.0 ± 1.1^∗^	74.0 ± 9.1^∗^	89.4 ± 1.5^∗^

Values are expressed as mean ± SD. ^∗^*p* < 0.05 for 5 Gy+DMSO vs. 5 Gy+vehicle; ^##^*p* < 0.01 for 5 Gy+vehicle vs. unirradiated; ^###^*p* < 0.001 for 5 Gy+vehicle vs. unirradiated; ^####^*p* < 0.0001 for 5 Gy+vehicle vs. unirradiated.

## Data Availability

The data used to support the findings of this study are available from the corresponding authors upon request.

## Supplementary Materials

Supplementary Materials Supplementary materials and methods: GC-1 and CT-26 cells and cell culture. Irradiation and DMSO administration of GC-1 and CT-26 cells. Cell Counting Kit-8 (CCK-8) assay of GC-1 cells. Apoptosis and ROS detection of GC-1 cells. Immunohistochemistry (IHC) and immunofluorescence (IF). Supplementary Table 1: the effect of DMSO on serum concentrations of sex hormones 45 days postirradiation. Supplemental Figure 1: DMSO radioprotects GC-1 cells. Supplemental Figure 2: DMSO has no radioprotective effect on CT-26 cells. Supplemental Figure 3: DMSO inhibits apoptosis of GC-1 cells after irradiation. Supplemental Figure 4: DMSO attenuates oxidative stress of GC-1 cells after irradiation.
