# Supplementary material for: Dimethyl Sulfoxide Attenuates Radiation-Induced Testicular Injury through Facilitating DNA Double-Strand Break Repair
Source: Oxid Med Cell Longev. 2022 Jun 20;2022:9137812. doi: 10.1155/2022/9137812 (PMC9236762; doi:10.1155/2022/9137812)
Supplement: Supplementary Materials — and methods: GC-1 and CT-26 cells and cell culture. Irradiation and DMSO administration of GC-1 and CT-26 cells. Cell Counting Kit-8 (CCK-8) assay of GC-1 cells. Apoptosis and ROS detection of GC-1 cells. Immunohistochemistry (IHC) and immunofluorescence (IF). Supplementary Table 1: the effect of DMSO on serum concentrations of sex hormones 45 days postirradiation. Supplemental Figure 1: DMSO radioprotects GC-1 cells. Supplemental Figure 2: DMSO has no radioprotective effect on CT-26 cells. Supplemental Figure 3: DMSO inhibits apoptosis of GC-1 cells after irradiation. Supplemental Figure 4: DMSO attenuates oxidative stress of GC-1 cells after irradiation. [file 9137812.f1.docx]

**Dimethyl Sulfoxide Attenuates Radiation-induced Testicular Injury Through Facilitating DNA Double-Strand Break Repair**

**SUPPLEMENTARY MATERIALS**

**Materials and Methods**

**Cells and cell culture**

The spermatogonia cell line GC-1 was purchased from the Procell Life Science＆Technology Co., Ltd (Wuhan, China). Cells were cultured in high-glucose dulbecco's modifed eagle's medium (DMEM; 8121431, Gibco, China) supplemented with 10% fetal bovine serum (aq 45586256, Analysis Quiz, China) and 1% penicillin-streptomycin (2321128, Gibco, China) and incubated in a humidifier incubator at 37˚C under 5% CO_2_.

The mouse colon carcinoma cell line CT-26 was purchased from Bioresource collection and Research Center (BCRC, China). Cells were cultured in Roswell Park Memorial Institute (RPMI) 1640 Medium (Gibco-Invitrogen, United States) supplemented with 10% FBS and 1% penicillin/streptomycin at 37°C under 5% CO_2._

**Irradiation and DMSO administration**

GC-1 cells were subjected to irradiation at a dose rate of 4 Gy/min and cultured with 0.1% DMSO (v/v) for 2 h prior to irradiation. DMSO was diluted in DMEM.

CT-26 cells were subjected to irradiation at a dose rate of 4 Gy/min and cultured with 0.5% DMSO (v/v) for 2 h prior to irradiation. DMSO was diluted in RPMI 1640.

**Cell Counting Kit-8 (CCK-8) assay of GC-1 cells**

GC-1 cells were plated in 96-well plates at a density of 1 × 10^3^ cells/well, cultured overnight and subsequently treated with 0.1% DMSO (v/v) for 2 h followed by 10 Gy, 12 Gy irradiation. CT-26 cells were plated in 96-well plates at a density of 2 × 10^3^ cells/well, cultured overnight and subsequently treated with 0.5% DMSO (v/v) for 2 h followed by 2, 4, 6 Gy irradiation. The CCK-8 (AQ308-500t, China) was used to assess the cells viability according to the manufacturer’s protocols.

**Apoptosis and ROS detection of GC-1 cells**

Apoptosis of GC-1 cells was measured using the Muse® Annexin V & Dead Cell kit (MCH100105; Luminex), following the manufacturer's instructions. Briefly, 1×10^5^ - 1×10^6^/mL single-cell suspension were prepared in advance, and 100 µl Muse Annexin V & Dead Cell Reagent was added to each tube and then incubated for 20 minutes at room temperature while protected from light. Then, the samples were analyzed by a Guava® Muse Cell Analyzer.

ROS levels in GC-1 cells were assayed by a Muse Oxidative Stress kit (MCH 100111, Luminex). Cell samples (1×10^5^/mL) and Muse Oxidative Stress Reagent solution were prepared with 1X Assay buffer, mixed thoroughly, and then incubated for 30 minutes at 37 °C in the dark. The number of ROS-positive cells was measured in a Guava® Muse Cell Analyzer.

**Immunohistochemistry (IHC)**

For the IHC analysis, the cross-sectioned testis was embedded in paraffin and cut into 4-μm- thick sections. After deparaffinization, testicular sections were subjected to antigen repair and blocked in 5% goat serum for 1 h at room temperature. Then, tissue sections were incubated at 4°C overnight with primary antibodies: c-Kit antibody (ab25634, Abcam, 1:1000); cleaved caspase-3 antibody (9664S, Cell Signaling Technology, 1:200); GFRα-1 antibody (sc-271546, Santa Cruz, 1:50); Ki67 antibody (ab16667, Abcam, 1:200); DDX4/MVH antibody, (ab13840, Abcam, 1:200); γ-H2AX antibody, (05-636, Merck Millipore, 1:1000). The number of stained cells was quantified for at least 30 seminiferous tubules per slide.

**Immunofluorescence (IF)**

For γ-H2AX IF of testicular tissue, the procedure was similar to immunohistochemistry except for the secondary antibody: Goat anti–mouse Alexa Fluor 488 (A0428, Beyotime, China).

For IF of GC-2 cells, cells were seeded onto glass bottom cell culture dishes (801002, NEST, China) and either left pretreated with 0.5% DMSO (v/v) or subjected to 10 Gy of radiation. At various time points after IR (0.5 h, 1 h, 6 h, 12 h, 24 h, 48 h), the GC-2 cells were fixed with 4% paraformaldehyde, permeabilized with 0.2% Triton X-100 in TBST and blocked in 5% goat serum, followed by staining with γ-H2AX (Merck Millipore, 05-636, 1:2000). Goat anti–mouse Alexa Fluor 488 (A0428, Beyotime, China) was used for secondary staining, followed by DAPI (C1002, Beyotime, China) counterstaining. Images were taken on a confocal microscope under 1000× magnification (LSM-700, ZEISS, Germany). The γ-H2AX positive foci were analyzed from the images of IF staining by using Image J software 8.0 (National Institutes of Health, USA).

| **Supplementary Table 1. The effect of DMSO on serum concentrations of sex hormones 45 days post-irradiation** | | | | | | |
| --- | --- | --- | --- | --- | --- | --- |
| Group | TS (ng/ml) | LH (mIU/mL) | | | FSH (mIU/mL) | GnRH (mIU/mL) |
| Unirradiated | 53.7 ± 8.5 | | 15.0 ± 1.8 | | 147.2 ± 8.0 | 229.8 ± 19.5 |
| DMSO | 46.9 ± 3.2 | | 13.7 ± 0.8 | | 140.0 ± 7.0 | 193.0 ± 13.9 |
| 5 Gy + Vehicle | 22.6 ± 2.3^##^ | | 5.3 ± 0.1^###^ | | 63.4 ± 7.9^###^ | 126.7± 13.2^##^ |
| 5 Gy + DMSO | 30.4 ± 3.1^*^ | | 8.9 ± 0.4^***^ | | 86.7 ± 8.7^*^ | 95.8 ± 4.0^*^ |
| values are expressed as Mean ± SD | | | |  |  |  |
| ^*^*p* < 0.05 for 5 Gy + DMSO vs. 5 Gy + Vehicle; ^***^*p* < 0.001 for 5 Gy + DMSO vs. 5 Gy + Vehicle | | | | | | |
| ^##^*p* < 0.01 for 5 Gy + Vehicle vs. Unirraiated; ^###^*p* < 0.001 5 Gy + Vehicle vs. Unirraiated; | | | | | | |


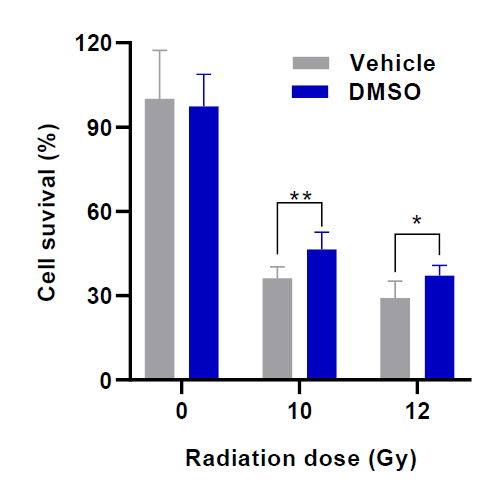


**Supplemental Figure 1. DMSO radioprotects GC-1 cells.** GC-1 cells were treated with 0.1% DMSO (v/v) or vehicle for 2 h prior to irradiation. Cell survival was detected by CCK-8 after 10 Gy and 12 Gy irradiation. Data from all irradiated samples were normalized to unirradiated samples, and percentages of viable cells were plotted. Error bars indicate mean ± SD, n = 3, **p* < 0.05; ***p* < 0.01. Student’s t tests were used to determine statistical significance. CCK-8, Cell Counting Kit-8.


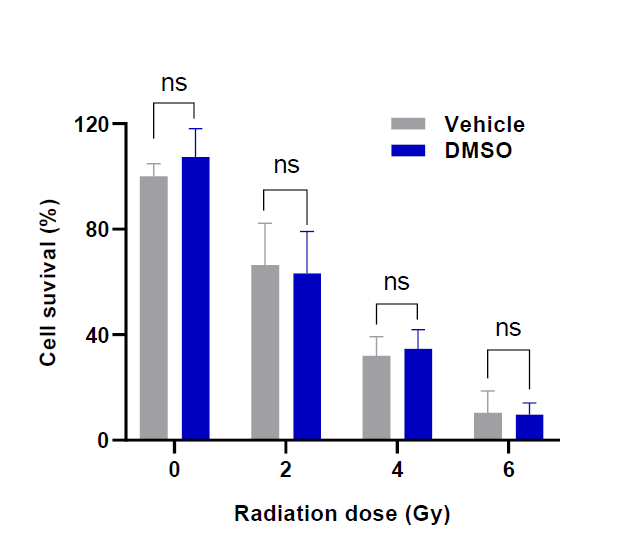


**Supplemental Figure 2. DMSO has no radioprotective effect on CT-26 cells.** CT-26 cells were treated with 0.5% DMSO (v/v) or vehicle for 2 h prior to irradiation. Cell survival was detected by CCK-8 followed by various doses (0, 2, 4, 6 Gy) of irradiation. Data from all irradiated samples were normalized to unirradiated samples, and percentages of viable cells were plotted. Error bars indicate mean ± SD, n = 3. Student’s t tests were used to determine statistical significance. ns, no significance.


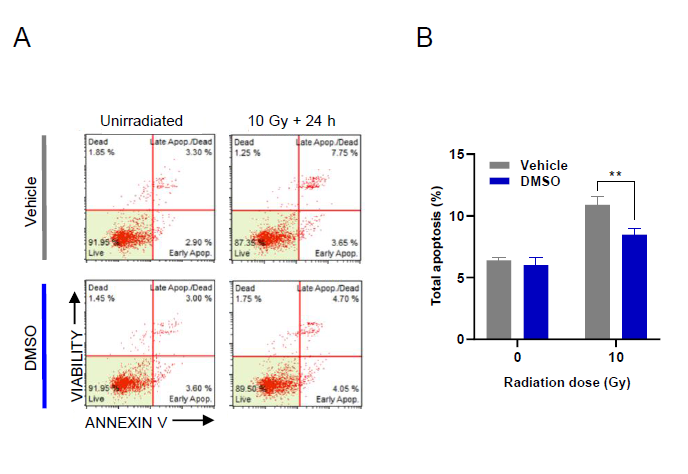


**Supplemental Figure 3. DMSO inhibits apoptosis of GC-1 cells after irradiation.** GC-1 cells were treated with 0.1% DMSO (v/v) or vehicle for 2 h prior to irradiation. (A) Representative flow cytometric analysis of apoptosis in GC-1 cells 24 h post-IR. (B) percentages of Annexin V positive GC-1 cells. Error bars indicate mean ± SD, n = 3, ***p* < 0.01. Student’s t tests were used to determine statistical significance in B.


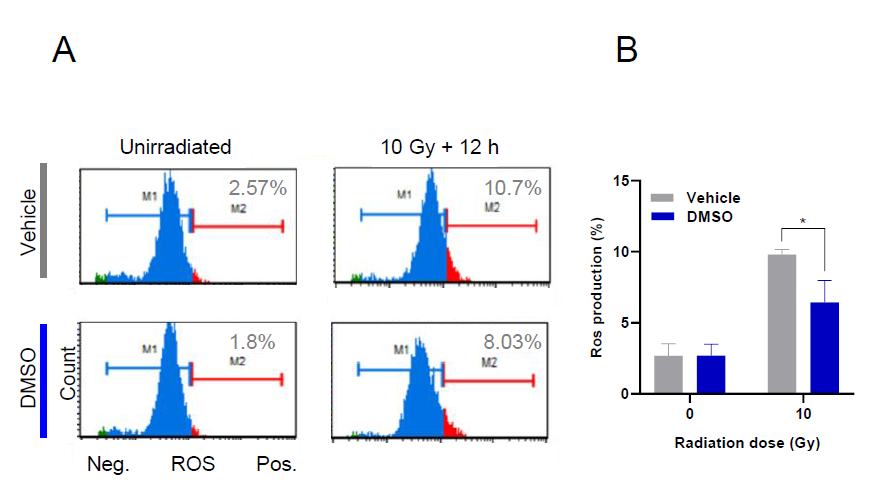


**Supplemental Figure 4. DMSO attenuates oxidative stress of GC-1 cells after irradiation.** GC-1 cells were treated with 0.1% DMSO (v/v) or vehicle for 2 h prior to irradiation. (A) Representative flow cytometric analysis of ROS in GC-1 cells 12 h post-IR. (B) Quantification of percentages of the ROS positive GC-1 cells. Error bars indicate mean ± SD, n = 3, **p* < 0.05. Student’s t tests were used to determine statistical significance in B.
